# [18F]FDG PET/MR to assess disease extension and inflammation in children and young adults with primary ciliary dyskinesia

**DOI:** 10.1007/s00259-025-07521-7

**Published:** 2025-08-30

**Authors:** Silvia Carraro, Valentina A. Ferraro, Pietro Zucchetta, Stefania Zanconato, Francesca Serani, Chiara Giraudo, Diego Cecchin

**Affiliations:** 1 Unit of Pediatric Respiratory Medicine and Allergy, Women’s and Children’s Health Department - SDB, University Hospital of Padua, Padova, Italy; 2 Unit of Nuclear Medicine, Department of Medicine -DIMED, University Hospital of Padua, Padova, Italy; 3 Unit of Advanced Clinical and Translational Imaging, Department of Cardiac, Thoracic, Vascular Sciences and Public Health – DCTV, University Hospital of Padua, Padova, Italy

**Keywords:** Primary ciliary dyskinesia, [18F]FDG PET/MR, HRCT, Inflammation, children

## Abstract

**Background:**

Primary ciliary dyskinesia (PCD) is a rare condition characterized by ciliary dysfunction, impaired mucociliary clearance and mucus accumulation in the airways.

**Purpose:**

Our aim was to evaluate the performance of [18F]FDG PET/MR in assessing structural and inflammatory pulmonary features in patients with PCD, using high-resolution CT (HRCT) as the gold-standard reference.

**Materials and methods:**

We recruited patients with PCD (≥ 7 years) regularly followed at our Regional Center for PCD. They underwent chest HRCT and [18F]FDG PET/MR using sequences optimized for the morpho-functional study of the lung. Parametric PET images were obtained by dividing each voxel by the mean value in a reference area. The volume of interest (VOI in cm^3^), named Metabolic Inflammatory Volume (MIV), was calculated by thresholding the PET parametric image using a value twice the mean of the reference area. Standardized Uptake Value (SUV) Max, SUV mean, Total Lesion Glycolysis (TLG) and MIV were recorded. HRCT and MR were analyzed using the Eichinger score.

**Results:**

Sixteen patients were enrolled. The Bland-Altman plot showed good agreement between HRCT and MR scores. Cumulative HRCT and MR scores correlated significantly with SUV mean score (HRCT: *p* = 0.02, r_s_=0.6; MR: *p* = 0.006, r_s_=0.66) and MIV (HRCT: *p* = 0.003, r_s_ =0.7; MR: *p* = 0.004, r_s_ =0.69). Total HRCT and MR scores and MIV score inversely correlated with spirometric parameters.

**Conclusion:**

PET/MR proved to be accurate in evaluating disease extent in PCD. It enabled the simultaneous assessment of structural damage and lung inflammation, both of which resulted inversely related to lung function. PET/MR is a promising tool for PCD monitoring.

## Introduction

Primary ciliary dyskinesia (PCD) is a genetically heterogeneous condition, autosomal recessive or X-linked, characterized by ciliary dysfunction and impaired muco-ciliary clearance. Affected patients present with chronic suppurative disease of the upper and lower airways. The main respiratory clinical features include a year-round daily wet cough, rhinosinusitis and recurrent acute infections [1].

Radiological studies based on high-resolution computed tomography (HRCT) have demonstrated that PCD is characterized by a predominance of bronchiectasis in the middle and lower lobes, diffuse tree-in-bud nodules, mucus plugging and atelectasis [2].

HRCT remains the gold standard for investigating and monitoring lung structural features in PCD patients over time. Nevertheless, there is a growing interest in the scientific community regarding the potential application of magnetic resonance (MR). Several studies have assessed its role in patients with cystic fibrosis (CF), a condition with similar, though usually more severe, pulmonary involvement. Given the technological advancements of the last decade, this radiation-sparing approach can now be considered a complementary technique and/or an alternative to HRCT for the long-term monitoring of cooperative CF patients [3].

Similarly, MR has also been proposed for pulmonary assessment in patients with PCD, demonstrating that structural lung damages can be effectively investigated using this imaging technique [4, 5].

Like other chronic suppurative lung diseases, PCD is characterized by chronic neutrophilic inflammation of the lung. The degree of inflammation can be estimated measuring inflammatory cells and mediators in bronchoalveolar lavage or sputum [6], but these techniques provide no information on the anatomical distribution of inflammation. On the other hand, nuclear medicine techniques, such as PET/CT and PET/MR, can offer valuable insights into the distribution of inflammation.

Few studies have explored the potential role of PET/CT in CF patients to quantify lung inflammation and assess changes in response to antibiotic therapy [7, 8]. However, to the best of our knowledge, no similar studies have been conducted in PCD patients.

In this study we applied, for the first time, the multi-modal imaging [18F]FDG PET/MR in children and adolescents with PCD. Our aims were: (1) to assess the performance of MR compared to HRCT in lung morphological assessment; (2) to evaluate the performance of PET in assessing pulmonary inflammation.

## Materials and methods

### Study design

Patients with PCD aged seven years or older followed at our Regional Center for PCD were recruited between June 2021 and July 2022. Recruitment was performed when patients -according to reported symptoms and physical examination - were clinically stable, at least one month after a pulmonary exacerbation defined according to Lucas et al. [9] Medical records were reviewed to collect demographic data and clinically relevant data.

At recruitment patients underwent a complete physical examination and performed spirometry. Chest HRCT and [18F]FDG PET/MR were conducted within 15 days of each other. Additionally, a sputum culture was performed.

The study was approved by the Ethic Committee of Padova (protocol CESC: 4818/AO/20; URC: AOPI724); parents and, when applicable, participants provided written informed consent.

### Spirometry

Spirometry was performed using a 10-L bell spirometer (Biomedin, Padova, Italy). The spirometer’s calibration was checked weekly with a 3-L syringe (Biomedin Padova, Italy). Forced vital capacity (FVC), forced expiratory volume in 1 s (FEV_1_), and forced expiratory flow rate at 25–75% of FVC (FEF_25–75_%) were measured according to international ATS/ERS recommendations [10].

### HRCT technique and assessment

Each patient underwent a chest HRCT using a standardized protocol with a slice thickness of 1.5 mm on a 64-slices GE Scanner (Revolution Evo, General Electrics Healthcare Italy). A single radiologist (CG) with 15 years of experience in thoracic imaging assessed each HRCT applying the Eichinger score [11]. This score is based on the evaluation of bronchiectasis/bronchial wall thickening, mucus plugging, abscess/sacculation, and consolidation. The extent of each feature was rated as follows: 0 = absent, 1 = present and affecting ≤50% of the lobe, 2 = present and affecting > 50% of the lobe. ^11^ Since patients with PCD may have situs viscerum inversus [1], to avoid generating mismatches in the overall disease extension assessment we did not perform a side specific sub-analysis for either HRCT or MR images (see below).

### [18F]FDG PET/MR

#### PET technique and assessment

[18F]FDG PET/MR acquisitions were performed using a 3 T Biograph mMR scanner (Siemens, Erlangen, Germany). In accordance with the European Association of Nuclear Medicine guideline, patients were instructed to fast for at least six hours to minimize insulin and glucose levels before receiving an intravenous injection of 3 MBq/Kg of [18F]FDG. Following the injection, patients were instructed to rest for 60 min to optimize tracer uptake. After voiding the bladder and positioning a dedicated radiofrequency body and head-neck coil, PET/MR images of chest/lung were acquired. The dedicated MR lung protocol is described below.

PET data were reconstructed using 3D ordered subsets expectation maximization (OSEM) iterative algorithm with 3 iterations, 21 subsets and a 4-mm Full Width at Half Maximum (FWHM) Gaussian filter.

As it is well known, attenuation correction in PET/MRI is not performed through direct measurement (as in the case of PET/CT), but rather through segmentation methods based on specific MRI sequences such as DIXON, UTE, or CAIPIRINHA. In tissues with low MR signal, such as the lungs, the µ-map derived from segmentation can be affected by significant bias, which may lead to inaccurate PET quantification. In line with this assumption, an accurate visual analysis of attenuation MR μ-maps revealed significant biases in the lung and thoracic tissues, which could have affected semi-quantitative analysis, particularly in younger children. For this reasons, semiquantitative parameters were evaluated on non-attenuation corrected (NAC) PET images.

To further reduce quantification biases, two expert pediatric nuclear physicians (DC, PZ) with more than 20 years of experience blindly performed an internal normalization of PET data using PMOD software (PMOD Technologies LLC Industriestrasse, Faellanden, Switzerland). For each patient, a reference lung area unaffected by pathology (both on PET and MR) was identified. The area selected as reference was also checked on HRCT scan and considered not affected by disease. The size of the area was chosen on a case-by-case basis (generally choosing the apical lung regions if not affected by pathology) in order to avoid non-specific uptake of the chest wall while ensuring a size that would provide a reliable mean value. PET parametric values used for statistical analysis were obtained by dividing the data of each voxel affected by pathology on the NAC images by the mean value obtained in the reference area. The volume of interest (VOI) was then determined by thresholding the PET parametric image using a value twice the mean of the reference area [12]. The following parameters were recorded: Standardized Uptake Value (SUV) Max, SUV mean, Total Lesion Glycolysis (TLG), and the volume (cm^3^) of the VOI representing the Metabolic Inflammatory Volume (MIV), a novel metric introduced in this study in analogy with the Metabolic Tumor Volume (MTV) commonly used in oncology [13]. Furthermore, we calculated the percentage MIV (MIV %) relative to total lung volume segmented using PMOD.

#### MR sequences and assessment

The MR protocol for the PET/MR scan included a coronal T1-weighted (T1w) fast low-angle shot three dimensional (fl3D) spiral volumetric interpolated breath-hold examination (VIBE Siemens Research Software Package) with 1.25 mm slice thickness, 0.05 ms echo time (TE), 3.47 ms repetition time (TR), 5° flip angle and a T2w Balanced Steady State Free Precession Line Acquisition with Undersampling (BLADE) fat saturated sequence, with 5 mm slice thickness,126 ms TE, 5632 ms TR, and 120° flip angle.

Both sequences were used to assess disease extension using the previously mentioned Eichinger score [11]. MR and HRCT images were blindly evaluated by the same radiologist.

### Statistical analysis

Descriptive statistics was used to report demographic and clinical features of recruited patients. To assess the agreement between HRCT and MR scores Bland & Altman analysis and the Cohen’s k were applied, while to assess the inter-rater agreement for PET MIV scores intraclass correlation coefficient (ICC) was used. The Spearman correlation coefficient was used to analyze the relationship between variables. Group comparisons were performed using the Kruskal-Wallis and Mann-Whitney tests. The significance level was set at *p* < 0.05. All analyses were performed using the software Prism (version 10.3.0) (GraphPad software, Boston, MA).

## Results

16 children, adolescents and young adults were included in the study. The clinical characteristics of the patients are summarized in Table 1.

**Table 1:** Clinical features of the recruited PCD patients

Variable	Value
Age (median, range)	13.6 years (8–25)
Number (females)	16 (10)
Dextrocardia (number (prevalence))	5 (31%)
Respiratory distress at birth (number (prevalence))	10 (63%)
Chronic cough (number (prevalence))	14 (88%)
Chronic rhinosinusitis (number (prevalence))	12 (75%)
Positive sputum culture (number (prevalence))	6 (38%)
FEV_1_%pred (mean (SD))	75.6 (18.4)
FVC %pred (mean (SD))	85.5 (16.6)
FEF_25−75_%pred (mean (SD))	64.6 (25.5)
FEV1/FVC % (mean (SD))	92.8 (8.7)

### HRCT and MR imaging assessment

The Eichinger score was first evaluated in each lobe, showing significantly different values among lobes (*p* < 0.0001 for both HRCT and MR). In particular, the score of upper lobes (HRCT: 0.0 (0.0-1.75); MR: 0.0 (0.0-1.75)) was lower compared to that of lower lobes (HRCT (3.0 (1.25-6.0); MR (2.5 (1.0–5.0)) and that of middle lobe/lingula (HRCT 5.0 (3.0-6.75); MR 4.0 (3.25–6.75)).

Then, the total lung score for both HRCT and MR was calculated, and it is summarized in Table 2.

**Table 2:** HRCT and MR-based total lung scores

	HRCT score (median and IQR)	MR score (median and IQR)
Bronchiectasis	3.5(2.3-6.0)	3.0 (2.0-5.8)
Consolidations	2.0 (0.0-3.8)	2.0 (0.0–3.0)
Mucus Plugs	2.0 (0.25–3.8)	2.0 (0.25–3.8)
Tree-in-bud	1.5 (0.0-2.8)	0.0 (0.0–2.0)
**Total score**	8.5 (5.5–14.0)	8.0 (5.0–13.0)

**Fig. 1:**
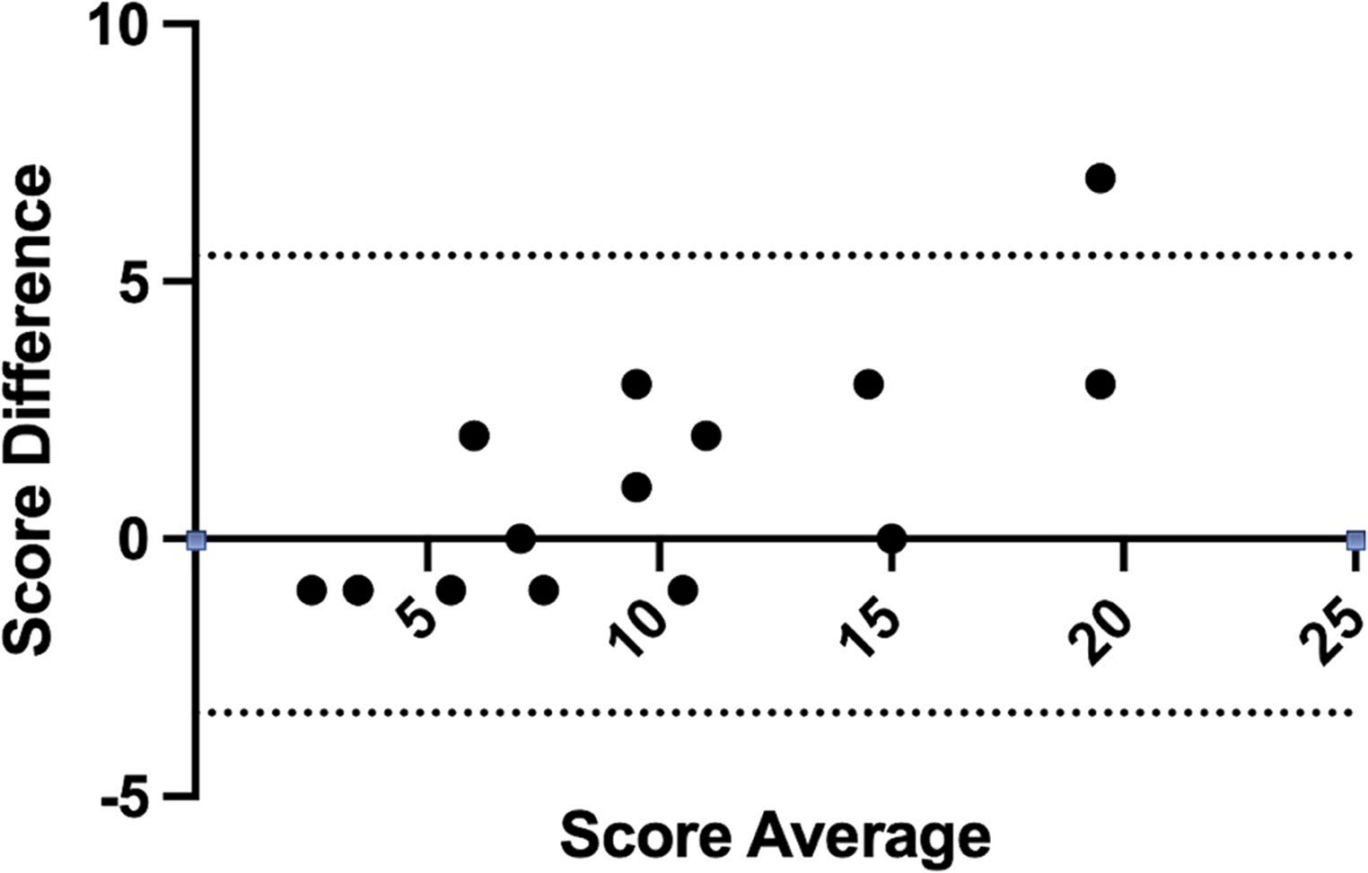
Bland Altman plot comparing total scores calculated on HRCT and MR

The Bland-Altman plot (Fig. 1) comparing the total score obtained with HRCT and MR showed a good agreement with a low bias (mean of the differences: 1.06). All but one data point fell within the 95% confidence interval. Similar results were found considering each feature (mean of the differences equal to 0.25 for bronchiectasis, 0.19 for consolidations, 0.06 for mucus plugs, 0.56 for tree-in.

-bud). In addition, the Cohen’s k between CT and MR showed a perfect agreement (k = 1) for consolidation and mucus plug scores and a substantial agreement (k = 0.6) for the tree in bud feature. As for bronchiectasis the Cohen’s k was not calculated because all the scans scored at least 1 on this parameter (i.e. we had no absence of this feature).

### PET assessment

PET scores are summarized in Table 3.

**Table 3:** PET scores: MIV (Metabolic inflammatory Volume); MIV % (Percentage MIV relative to total lung volume); SUV (Standardized Uptake Value); TLG (Total Lesion Glycolysis)

	Median	IQR
PET MIV (cm^3^)	10.50	1.03–87.60
MIV %	0.40	0.10–4.30
SUV Max	3.25	2.40–4.20
SUV Mean	2.60	2.40–3.60
TLG	24.70	3.37–200.60

Being MIV a new proposed metric, an inter-reader agreement analysis was performed on data derived from blinded segmentation and analysis performed by two expert readers, showing excellent agreement between operators (ICC 0.89, 95% CI 0.73 to 0.96).

### Correlation between morphologic (MR and CT) and metabolic (PET) scores

The bronchiectasis score, as calculated on both HRCT and MR, significantly correlated with the PET MIV (*p* = 0.03, r_s_=0.56 [95%CI 0.08 to 0.83] and *p* = 0.009, r_s_ =0.64 [95%CI 0.19 to 0.86] respectively), while the correlation with the PET SUV mean was only marginally significant (*p* = 0.049, r_s_ =0.5 [95%CI −0.008 to 0.86] and *p* = 0.06, r_s_ =0.49 [95%CI −0.03 to 0.80] respectively)

Tree-in-bud score, as calculated on HRCT but not on MR scans, significantly correlated with MIV (*p* = 0.03, r_s_ =0.56 [95%CI 0.07 to 0.83]).

No correlation was found between the SUV mean, SUV Max or MIV and the mucus plug or consolidation scores on either MR or HRCT.

**Fig. 2:**
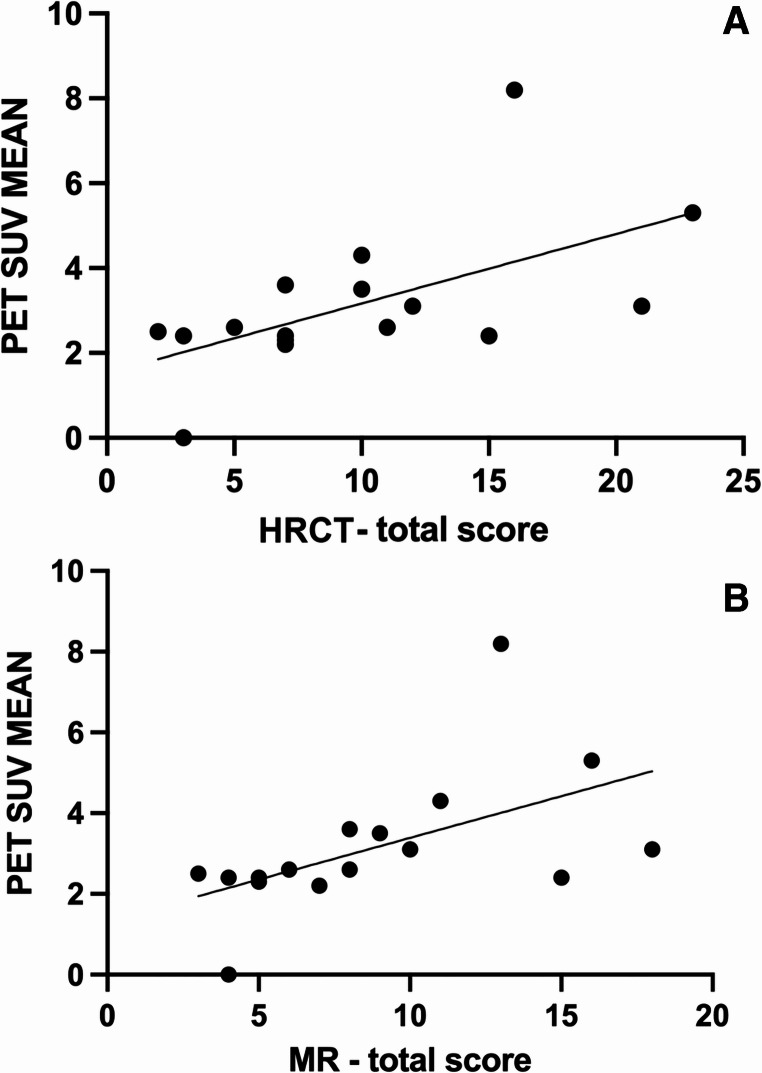
Correlation between PET SUV mean and HRCT total score (**A**) (*p* = 0.02, *r* = 0.6) and MR total score (**B**) (*p* = 0.006, *r* = 0.66)

When considering all features assessed on HRCT and MR as a measure of overall lung injury, cumulative scores significantly correlated with the SUV mean (Fig. 2) (HRCT cumulative score: *p* = 0.02, r_s_=0.6 [95%CI 0.14 to 0.85]; MR cumulative score: *p* = 0.006, r_s_=0.66 [95%CI 0.24 to 0.88]) and with MIV (HRCT cumulative score: *p* = 0.003, r_s_=0.7 [IC 0.31 to 0.89]; MR cumulative score: *p* = 0.004, r_s_=0.69 [95%CI 0.29 to 0.89]), and with TLG (HRCT cumulative score: *p* = 0.002, r_s_=0.71 [IC 0.32 to 0.90]; MR cumulative score: *p* = 0.003, r_s_=0.70 [IC 0.30 to 0.89]). No correlation was found with SUV Max.

### Correlation between imaging scores and clinical data

The SUV mean and the MIV scores did not differ between patients who experienced two or more pulmonary exacerbations in the previous year and those who had only one or no exacerbations (*p* = 0.74 and *p* = 0.95 respectively). Similarly, no significant difference was found between patients with and without a positive sputum culture performed within 15 days of the PET/MR (*p* = 0.89 and *p* = 0.60 respectively).

MR and HRCT scores also did not differ based on the number of exacerbations in the past year or the results of the more recent sputum culture (data not shown).

A significant inverse correlation was found between HRCT and MR scores and both FEV_1_ and FVC, while no significant correlation was found with FEF_25−75_. Regarding PET scores, a significant inverse correlation was found between MIV and FVC (Table 4).

**Table 4:** Correlations between HRCT and MR scores (calculated using Eichinger score) and spirometric parameters

	FVC % pred	FEV_1_% pred	FEF_25−75_% pred
**HRCT total score**	*p* = 0.002; r_s_=−0.73 [95%CI −0.90 to −0.35]	*p* = 0.02; r_s_=−0.58 [95%CI −0.84 to −0.10]	*p* = 0.16; r_s_=−0.37 [95%CI −0.74 to 0.17]
**MR total score**	*p* = 0.001; r_s_=−0.75[95%CI −0.91 to −0.39]	*p* = 0.02; r_s_ =−0.59 [95%CI −0.84 to −0.11]	*p* = 0.15; r_s_=−0.38 [95%CI −0.38 to 0.16]
**PET SUV MEAN**	*p* = 0.06; r_s_=−0.48 [95%CI −0.80 to 0.03]	*p* = 0.09; r_s_=−0.44 [95%CI −0.78 to 0.08]	*p* = 0.65; r_s_ =−0.12 [95%CI −0.12 to 0.41]
**MIV**	*p* = 0.017; r_s_=−0.60 [95%CI −0.85 to −0.13]	*p* = 0.11; r_s_=−0.41 [95%CI −0.75 to 0.11]	*p* = 0.50; r_s_=−0.18 [95%CI −0.62 to 0.34]
**TLG**	*p* = 0.017; r_s_=−0.59 [95%CI −0.84 to −0.12]	*p* = 0.015; r_s_=−0.61 [95%CI −0.85 to −0.14]	*p* = 0.31; r_s_=−0.27 [95%CI −0.68 to 0.28]

## Discussion

This is the first study demonstrating that [18F]FDG PET/MR can be successfully applied to assess patients with PCD, enabling the simultaneous evaluation of lung morphological damage and metabolic changes associated with inflammation.

Even if HRCT remains gold standard for evaluating structural lung alterations, our findings, demonstrating a good agreement between HRCT and MR scores, suggest that MR provides a reliable alternative for the follow up of PCD patients. Our results confirm and expand upon earlier studies showing the role of lung MR in children with CF and with non-CF bronchiectasis [14, 15]. Compared to HRCT, MR offers the significant advantage of eliminating radiation exposure. This is particularly important in children who face a long disease course ahead with the potential for high cumulative radiation exposure over their lifetime [16].

**Fig. 3:**
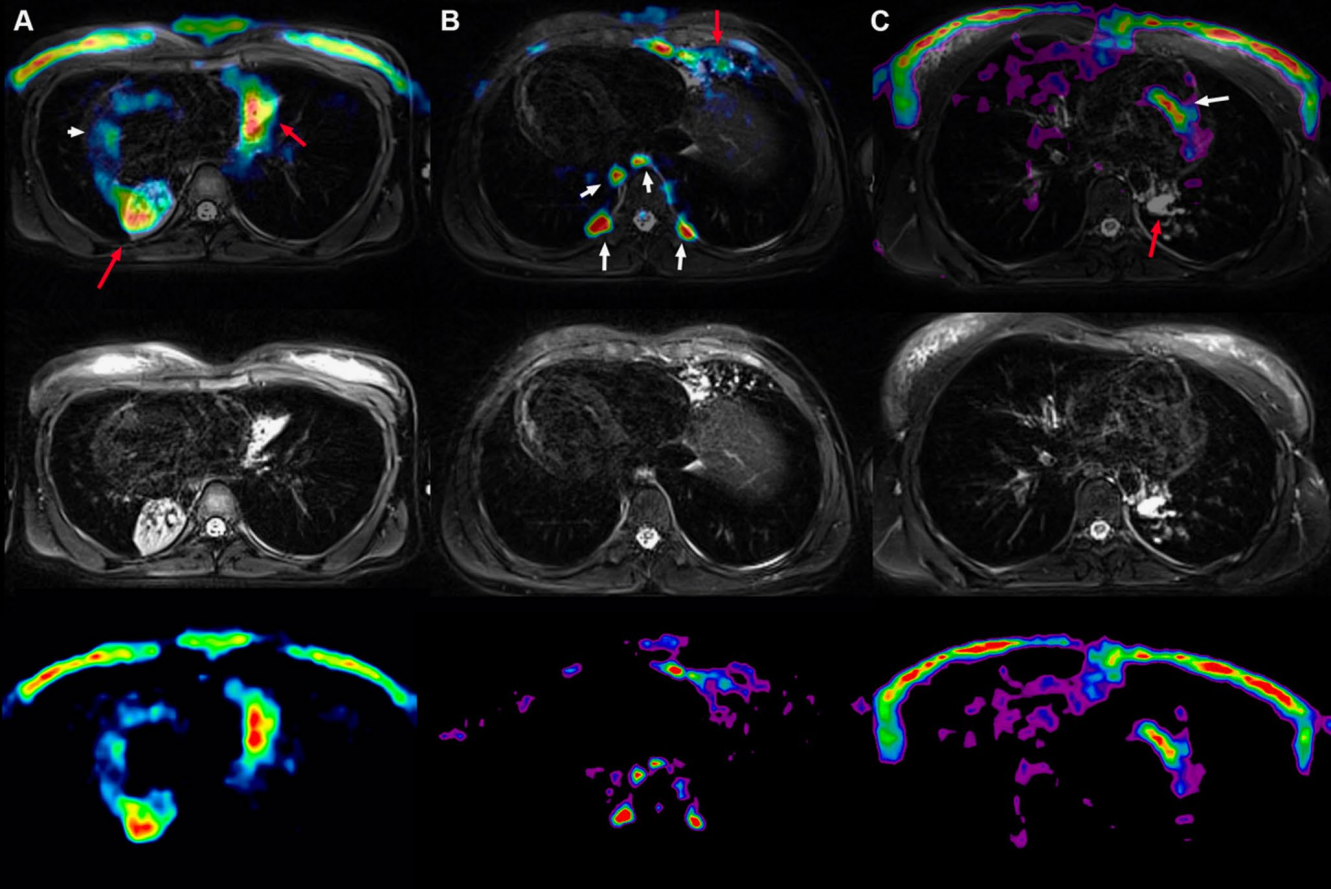
In the bottom row: parametric image (obtained from a PET not corrected for attenuation divided by the average count in a lung area not affected by the pathology). In the middle row: MR image (T2 blade fatsat respiratory triggered - TR:8830; TE:126; 1.18 × 1.18 mm). In the top row: fused image. In columns: three distinct patients: (**A**) female with dextrocardia showing high tracer metabolism in the para-cardiac areas (red arrows) corresponding to the alterations visible on MR and a non-specific cardiac uptake (white arrow); (**B**) male with dextrocardia showing intermediate uptake (red arrow) corresponding to the alterations visible on MR and a non-specific uptake corresponding to activation of paravertebral brown fat; (**C**) female showing absent metabolism corresponding to the areas of consolidation visible on MR (red arrow) and non-specific cardiac uptake (white arrow)

Examining the correlations between morphological features on HRCT and MR scans and inflammation on PET scans, we found some notable results. As expected, none of the morphological scores correlated with SUV Max, which represents the voxel with the highest [18F]FDG uptake, a parameter of limited relevance in PCD. Instead, the strongest correlations were observed with MIV, a parameter that accounts for both the intensity of metabolic activity and the volume of affected lung parenchyma, and with TLG (a combination of MIV and SUV mean). Derived from oncological studies [17] we propose that MIV and TLG may be appropriate metrics for capturing the inflammatory state in PCD lungs. In fact, MIV and TLG correlated significantly with both cumulative MR and HRCT scores, underscoring the close relationship between overall lung damage and ongoing inflammation. On the other hand, it is worth noting that some lung areas with significant morphologic alterations exhibited minimal or no active inflammation (Fig. 3) demonstrating that the metabolic PET alterations provide complementary information as compared to MR instead of merely reflecting the morphological changes. For this reason, monitoring PCD through a technique which provides both morphological and functional information, like PET/MR, can offer a more comprehensive assessment of the activity and distribution of lung disease. Only few studies evaluated lung inflammation in PCD [18, 19] and, so far, no biomarker has been proposed as gold standard to address this aspect of the disease. For this reason, we could not correlate PET data with other standardized measures of inflammation. Nonetheless our data together with previous studies showing, for example, increased levels of cytokines and proteases in sputum of PCD subjects [18, 19], support the key role of inflammation in PCD pathogenesis and prompt the need for assessing this disease dimension, beside the structural one. Understanding and quantifying inflammation, in fact, could pave the way to the development of anti-inflammatory therapies and to the possibility of monitoring their effects [20].

The importance of MIV is further supported by its significant correlation with bronchiectasis scores, on both HRCT and MR. Bronchiectasis, indeed, is a key morphological feature of PCD being present in about 50% of children by the age of 8 years and in nearly 100% of adults [21]. Notably, the correlation of MIV with the MR score was stronger than with HRCT, suggesting that MR, despite its lower anatomical resolution, may better reflect the inflammatory and functional aspects of bronchiectasis. The correlation of morphological scores with MIV highlights that bronchiectasis are affected by chronic inflammation and this is, indeed, a topic of growing interest also in the view of potential therapeutic interventions targeting specific inflammatory pathways [22].

An interesting correlation also emerged between the tree-in-bud pattern and MIV. The tree-in-bud pattern is indicative of endo-bronchiolar inflammation, and its positive association with MIV suggests that PET is sensitive enough to detect inflammatory processes in the peripheral airways [23] in PCD patients. An inverse correlation was recently reported between peripheral airway resistance measured by forced oscillatory technique (FOT) and quality of life (QoL), underscoring the clinical impact of smaller airway disease in PCD [24].

Interestingly, neither PET inflammatory metrics nor MR/HRCT structural scores were associated with the clinical course of the disease. Specifically, patients with more than 2 exacerbations in the past year or a positive sputum culture within 15 days of imaging tests did not exhibit significantly higher PET, MR or HRCT scores. However, imaging-derived pathological findings were significantly related to lung function, which is indeed an important clinical parameter. Considering the correlation with spirometry, we found a significant inverse correlation between both FVC and FEV_1_ and the cumulative lung damage measured via HRCT and MR score. Additionally, FVC was inversely correlated with MIV and both spirometric parameters were inversely correlated to PET TLG. These results confirm and expand upon the findings of Sagel et al. [19], who reported a correlation between inflammatory biomarkers in PCD sputum and both impaired lung function and structural lung damage.

A key limitation of our study is the small sample size, which limits the generalizability of our results. Moreover, because of the small sample size we cannot rule out the possibility that the lack of correlation in some areas (e.g. with exacerbations or microbiological results) is actually due to a lack of power. We acknowledge that being this limitation inherent to the study of PCD, a rare disease, in the context of a monocentric study, further multicentric studies are needed to confirm our results.

Another limit is the absence of a control group. Due to ethical considerations, healthy subjects could not be included, as performing these imaging tests in asymptomatic individuals would not be justified.

A further limit is that compared to MR alone, PET/MR, while providing important information on the inflammatory state, implies a certain degree of radiation exposure. The level of such exposure is indeed quite low: the estimated effective dose administered from the [18F]FDG PET injection was systematically under the 0.037 mSv/MBq (ICRP 128 Table C31) for children between 5 and 10 years and under the 0.024 mSv/MBq (ICRP 128 Table C31) for children between 10 and 15 years. Moreover, the new technologies and new generations of PET/MR that are coming out on the market promise to reduce the effective dose by a factor of at least 2 in the next future. In addition, although it is not possible to draw definitive conclusions that justify the use of PET/MRI over the use of PET/CT combined with MRI, we can nonetheless assume at least three advantages of PET/MRI: (1) a purely logistical benefit for families, related to the ability to complete the entire examination in a single day; (2) the possibility of performing a single sedation in children who require it; (3) the dose reduction due to the omission of the CT component present in PET/CT.

Finally, a limit of the study is that we did not use a tailored PCD score for MR and HRCT but we consider the Eichinger score appropriate since it was already applied on MR-based studies also for PCD patients. Other scores, such as the SPEC [25], could be considered in further studies.

## Conclusions

In the present study, MR enabled an accurate evaluation of lung structural damage, with a good agreement of the MR scores compared to those calculated on HRCT, which remains the gold standard. In addition, PET analysis proved to be effective in identifying presence and distribution of lung inflammation. In conclusion, PET/MR proved to be a valuable tool for assessing disease extent in PCD patients. Further multicentric studies are needed to confirm our data and to evaluate the possible role of PET/MR in longitudinal monitoring of PCD patients, including assessment during acute exacerbations.

## Data Availability

The datasets generated and analysed during the current study are available from the corresponding author on reasonable request.
